# Geochemical constraints and heritage conflicts during cadmium stabilization of Zn-Pb-Ag tailings at a UNESCO World Heritage site

**DOI:** 10.1038/s41598-026-48819-2

**Published:** 2026-04-17

**Authors:** Jerzy Cabała, Rafał Warchulski, Krzysztof Kupczak, Wojciech Kubica, Arkadiusz Krzątała

**Affiliations:** 1 Institute of Earth Sciences, Faculty of Natural Sciences, University of Silesia, Będzińska 60, Sosnowiec, 41-200 Poland; 2 Department of Solid Fuels Quality Assessment, Central Mining Institute - National Research Institute, Plac Gwarków 1, Katowice, 40-160 Poland

**Keywords:** Mining legacy, UNESCO World Heritage, Biochar chemostabilization, Cadmium mobility, Carbonate-hosted deposits, Sustainable remediation, Ecology, Ecology, Environmental sciences

## Abstract

Remediating historical mining waste creates a paradox when environmental safety conflicts with the preservation of the visual integrity of UNESCO World Heritage sites. This study characterizes the geochemical constraints on Zn-Pb-Ag tailings at the Tarnowskie Góry site (Poland), where strict conservation laws prohibit traditional capping methods. Using X-ray diffraction, SEM-EDS, and sequential extraction, we identified contrasting mobility patterns in potentially toxic elements. While Pb (up to 2.15 wt%) and Zn (up to 11.2 wt%) remain sequestered in stable phases, cadmium (up to 1020 mg kg^− 1^) exhibits lability, with up to 74% partitioned in exchangeable (up to 13%) and carbonate (up to 61%) fractions. Although aqueous leaching demonstrates negligible current mobilization, this partitioning poses a latent risk of release due to localized rhizosphere acidification or microenvironmental carbonate depletion. Furthermore, heritage status effectively restricts the potential extraction of an estimated 150 tons of Ag, 123,000 tons of Zn, and 19,800 tons of Pb. We propose a conceptual dual-zone sustainable management model: (1) Assisted phytostabilization using native calcicolous species for stable slopes; and (2) ‘invisible’ chemostabilization using Fe–modified biochar amendments for protected zones where vegetation would compromise historical industrial aesthetics. Engineered biochar effectively immobilizes labile Cd while preventing the secondary mobilization of background As, a risk typically associated with conventional biochar. Concurrently, it reduces wind erosion without altering the waste’s visual character. These findings provide a scalable conceptual framework for reconciling pollution control with the preservation of Outstanding Universal Value at carbonate-hosted mining legacies globally.

**Supplementary Information:**

The online version contains supplementary material available at 10.1038/s41598-026-48819-2.

## Introduction

Metal(loid)- rich waste repositories are a relic of past metal mining and smelting operations worldwide^1,2^. Potentially toxic elements (PTEs) have been released into the environment for centuries as a result of ore extraction and processing, dating back to antiquity and intensifying during the 19th– to 20th–century industrial boom^3–5^. These legacy mining and smelting wastes alter the soil and water chemistry, often causing PTE contamination that poses ecological and human health risks^6–9^. Elements such as Pb, Cd, As, and Zn from mine tailings and smelter slags can persist in soils for centuries, with adverse impacts on biota and public health^2,10,11^. The problem of such PTE pollution is especially significant in regions with a history of ore mining and processing^12^.

The Upper Silesian district of southern Poland is a perfect example of such a region, being one of Europe’s oldest and largest base metal mining areas, where centuries of Pb–Zn–Ag extraction and smelting have caused severe contamination of soils, waters, and biota^13–15^. Such contamination can result in elevated PTE concentrations in crops and wildlife, as well as increased exposure risks for local populations^16,17,8,18^. Studies have shown that historical mining pollution can be linked to contemporary PTE bioavailability and health hazards, even many years after the cessation of activities^3,19,20^. This underlines the need for comprehensive mineralogical and geochemical characterization of historical mining wastes^21,22^.

Identifications of the PTE-hosting phases together with their bulk concentration provide a basis for the prediction of how these elements may be released or immobilized under varied environmental conditions^23,24^ and estimation of their potential toxicity^6,2^. Elements such as Pb, Cd, As, and Tl are of particular concern due to their toxicity at low concentrations and their tendency to accumulate in food chains^10,12^. Understanding the total load and distribution of these PTEs in the tailings is crucial for environmental risk assessment^8^. Additionally, mineralogical and geochemical characterization of tailings can reveal the presence of residual valuable metals. In the case of Tarnowskie Góry, historical records indicate that silver was a primary commodity (with lead and zinc) since the Middle Ages. However, the actual Ag content retained in the discarded tailings has not been documented. Quantifying residual Ag and other metals (e.g., Zn, Pb, Fe) can provide information about the past processing efficiency and the resource recovery potential of the waste heap^1^.

The present study was undertaken to fill these knowledge gaps by conducting an in-depth mineralogical and geochemical analysis of historical ore-processing and smelting waste at the UNESCO Tarnowskie Góry site. We analyzed the phase composition of Zn–Pb–Ag tailings and determined the concentrations of PTEs (As, Cd, Pb, and Zn) in the waste materials. Based on the occurrence of PTE-bearing minerals and considering their operational mobility in sequential leaching tests, we aim to evaluate the potential for these legacy wastes to release contaminants into the environment. The results provide insight into whether the old tailings pile currently serves as a source of PTEs to soils and waters, or whether natural attenuation (e.g., formation of stable secondary minerals) limits their mobility (e.g.^25–27^. Additionally, the study estimates the total stock of PTEs concentrated in the waste heap, which has implications for both environmental management and resource accounting.

However, managing such historical post-industrial landscapes creates a unique challenge. Considering that heritage sites like Tarnowskie Góry are legally protected for their historical value, traditional remediation or re-exploitation is restricted. This creates a unique challenge: the historical post-industrial landscape must be managed to mitigate environmental hazards without compromising its cultural heritage^22^. By characterizing the tailings’ composition and PTE behavior, the study helps guide the safe stewardship of the Tarnowskie Góry site. Together, our findings enhance understanding of PTE mobility and the potential for metal recovery in legacy mining wastes, and propose conceptual frameworks for balancing environmental remediation with the conservation of historically important industrial sites^21^.

### Historical background and geological setting

**Fig. 1:**
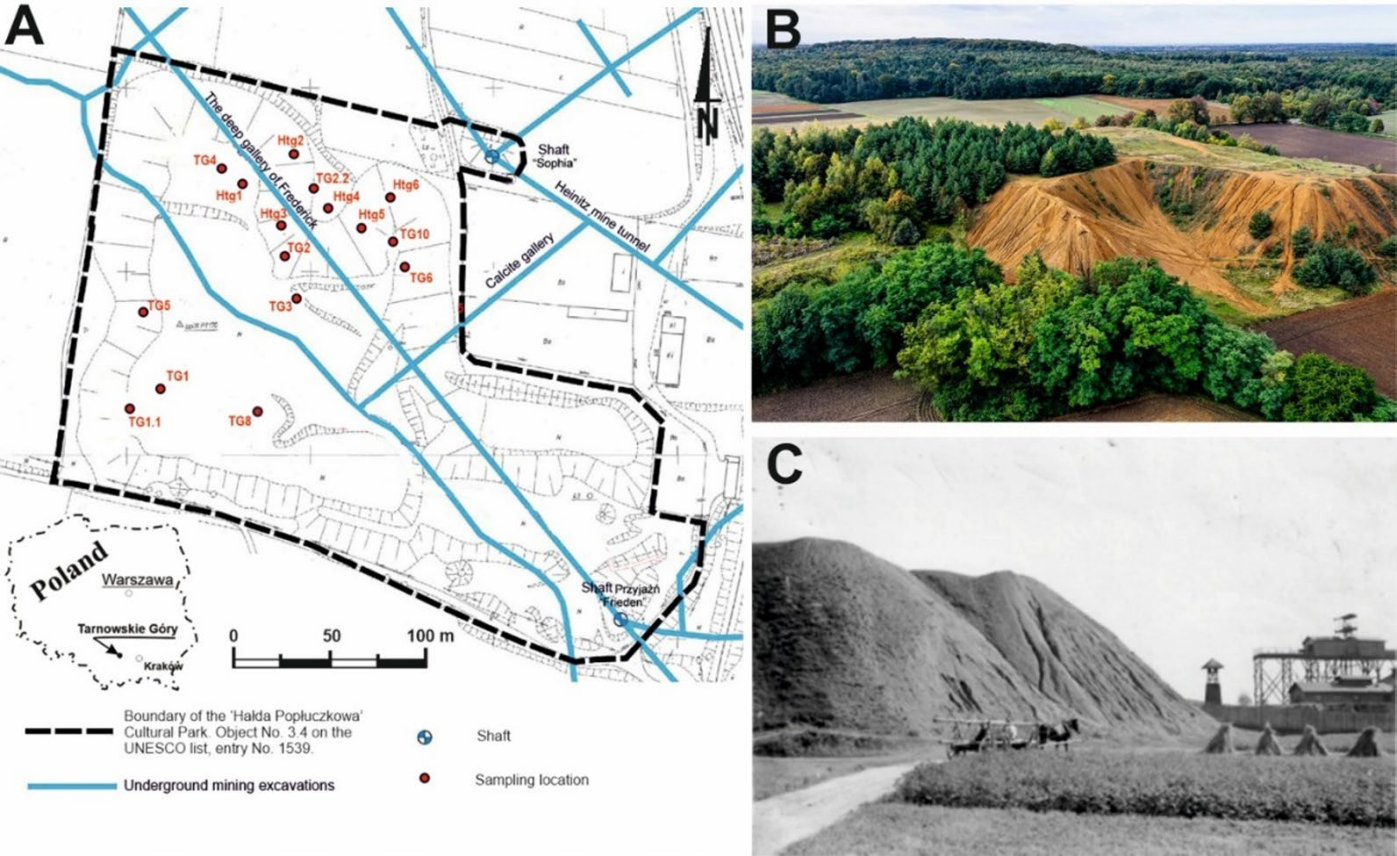
(**A**) Schematic of the historical Zn-Pb-Ag ore-washing site. The layout of the slopes and underground galleries is based on the UNESCO Nomination Text (Poland, UNESCO list, 2017). (**B**) View of the ore-washing waste site. Source: unesco.tarnowskiegory.pl. (**C**) The ore-washing waste pile in Tarnowskie Góry (Photo: Archive of the Tarnogórska Land Lovers Association). Source: unesco.tarnowskiegory.pl.

The Silesian-Cracow region (southern Poland) contains some of Europe’s largest Pb–Zn–Ag ore deposits, which have been continuously exploited since at least the 12th century^28^. Over 800 years of mining in this region have generated numerous dumps of metal(loid)-rich tailings and smelting residues scattered across the landscape^29^. The Tarnowskie Góry mining district in Upper Silesia, where extensive Pb, Ag, and Zn mining has left a significant footprint, is a prime example of such a site. Mining in Tarnowskie Góry dates back to the late Middle Ages (14th century) and by the modern era had created vast underground networks of shafts and drainage tunnels, along with substantial surface waste deposits. In recognition of its unique mining heritage, including an extensive underground water management system, Tarnowskie Góry was inscribed on the UNESCO World Heritage List in 2017 (Poland, UNESCO list^30^). A key component of the designated site is a historic waste pile covering approximately 0.5 km². This feature, reaching a height of roughly 17 m, contains an estimated 1.78 million tonnes of Zn-Pb ore-washing waste deposited between 1840 and 1912. Among the protected elements, this massive accumulation primarily originates from 19th-century Pb–Ag–Zn operations (Fig. 1). The operation of a Zn-Pb ore washing plant typically begins with the removal of barren dolomite and limestone aggregates. The process involves crushing and screening, followed by a washing stage utilizing drums or screens. This step removes clay and fine particles prior to further processing, such as smelting or flotation.

The Silesian-Cracow Zn-Pb-Ag deposits are classified as Mississippi Valley-type (MVT) deposits^31^. The ores are hosted by carbonate formations, primarily epigenetic Middle Triassic ore-bearing dolomites^28^. In the Tarnowskie Góry district, ore-bearing horizons and nests occur at shallow depths (20–60 m) and are locally exposed at the surface. Dolomites, limestones, and clayey-dolomitic strata of Middle and Upper Triassic age overlie the deposits. In places, these are covered by residual weathered sediments or Pleistocene-age fluvioglacial sands. Rendzina soils have developed on carbonate rocks, whereas podzolic soils predominate on sandy substrates^32^.

Primary mineralization is dominated by sulfides of Zn, Fe, and Pb, with Cd occurring less frequently. Subsequent weathering during the Miocene and Pleistocene led to the geochemical alteration of primary ores and the formation of supergene Zn-Pb-Fe accumulations^33,28^. Supergene assemblages comprise carbonates, sulfates, silicates, oxides, and hydroxides of the same metals^28^. In addition to Zn, Pb, and Fe, notable concentrations of Cd, Tl, As, Ag, and Mn (Table 1) are present in both primary and secondary ore minerals as well as in the associated gangue of the Silesian-Cracow deposits.

**Table 1:** Concentrations of Cd, Tl, As, and Mn in rocks and minerals associated with MVT deposits of the Silesian-Cracow area.

Element	Concentration	Material
Cadmium	up to 10,000 mg kg^− 131,34^	Sphalerite
	1,283–5,850 mg kg^− 128^	Ores
	> 1,000 mg kg^− 128^	Gangue
Thallium	up to 500 mg kg^− 135^	Sphalerite
	100–1,000 mg kg^− 134,31^	Sphalerite + Fe sulfides
	< 1,000 mg kg^− 128^	Marcasite ores
	< 90 mg kg^− 128^	Sphalerite ores
Arsenic	up to 20,000 mg kg^− 128^	Marcasite ores
	up to 10,000 mg kg^− 131^	Galena + Fe sulfides
Manganese	up to 10,000 mg kg^− 128^	Ores enriched in clay minerals

### Methods

#### Sampling

As part of the landfill site sampling program, two field campaigns were conducted. The first campaign involved collecting ten samples (TG1; TG1.1; TG2; TG2.2; TG3; TG4; TG5; TG6; TG8; TG10) of ore-washing tailings from across the entire landfill area (Fig. 1). These samples were subjected to bulk chemical analysis for trace elements to identify the main PTEs present at the site and to select the location for the second sampling campaign.

The second sampling campaign (Fig. 1) was conducted in the north-western part of the heap due to the high concentrations of PTEs identified during the preliminary survey and the limited vegetation cover, which could potentially influence material properties. This targeted sampling aims to characterize the most acute, highly exposed surface zones where direct environmental interaction is currently unmitigated. However, because sampling was restricted to specific surficial exposures, the acquired bulk geochemical parameters cannot be uniformly generalized to the full, unweathered subsurface deposit. Samples (Htg 1–6) were collected each time from a depth of approximately 20 cm. Each sample was analyzed for bulk chemical composition using XRF (X-ray Fluorescence), ICP-ES (Inductively Coupled Plasma Emission Spectrometry) and ICP-MS (Inductively Coupled Plasma Mass Spectrometry), as well as for phase composition using XRD (X-ray Diffraction) and for phase chemistry using SEM-EDS (Scanning Electron Microscopy coupled with Energy Dispersive Spectroscopy).

#### Phase composition

Backscattered-electron (BSE) Images and Energy-Dispersive Spectroscopy (EDS) analyses were conducted using a Thermo Scientific Quanta 250 scanning electron microscope (SEM) equipped with a 4-quadrant BSE semiconductor detector and an UltraDry EDS detector. Operating conditions were maintained under a high vacuum and with an accelerating voltage of 15 kV. The samples were crushed and sieved, and the < 0.2 mm fractions were prepared for SEM imaging and EDS microanalysis. Samples were affixed to aluminum stubs using double-sided carbon tape. Forty SEM slides of ore-washing tailings were analysed. BSE images and EDS microanalyses were made. Submicroscopic studies were carried out in the Microscopic Research Laboratory at the Faculty of Natural Sciences of the University of Silesia in Katowice.

X-ray powder diffraction data were obtained using a PANalytical X’PERT PRO–PW 3040/60 diffractometer (Malvern Panalytical Ltd, Malvern, UK) (CuKα source radiation, Ni-filter to reduce the Kβ radiation, and X’Celerator detector). Quantitative data processing was performed by means of the X’PERT High Score Plus software using the latest PDF4 + database. The research was carried out at the X-ray Structural Research Laboratory at the Faculty of Natural Sciences of the University of Silesia in Katowice.

#### Chemistry

The samples were first crushed using a jaw crusher and subsequently pulverized in an agate ball mill to produce a fine, homogeneous powder. This preparation method was selected to minimize metal contamination prior to geochemical analysis. Major oxides were determined using the XF700 package at Bureau Veritas Commodities Canada Ltd. For this analysis, the prepared pulp was mixed with a lithium borate flux (Li_2_B_4_O_7_/LiBO_2_) and fused in a furnace to form a glass bead. The resulting discs were analyzed using wavelength-dispersive X-ray fluorescence (WD-XRF). Loss on Ignition (LOI) was determined gravimetrically by roasting a separate aliquot. Additionally, total carbon (TOT/C) and total sulfur (TOT/S) concentrations were quantified using the TC000 package. This method involves the automated combustion of the sample in an induction furnace, followed by detection using infrared spectroscopy. For trace element analysis, approximately 0.25 g of each sample was digested using the four-acid method (HF + HClO_4_ + HCl + HNO_3_) with the use of thermal conductivity. Elements in these solutions were analysed by ICP-ES or ICP-MS , depending on the concentration of elements in the samples. ICP-ES analyses were performed by Bureau Veritas Commodities Canada Ltd. using the MA200 and MA270 analytical procedures. This methodology was chosen because of the low lower limits (in mg kg^− 1^) of quantification of, e.g., Cu (0.1), As (0.2), Cd (0.02), Sb (0.02), Tl (0.05) and Ag (20 µg kg^− 1^), however, the and Zn is high (10,000 mg kg^− 1^) upper limit of quantification for Pb. Quality assurance and quality control (QC/QA) were based on the certified reference materials (OREAS605, OREAS927-AR, OREAS927-MA, OREAS135, OREAS184, SY-3(D), GS311-1, GS910-4, GS314-1, GBM398-4-MA), procedural blanks, and sample duplicates. All geochemical results, alongside QA/QC data, are included in Tables S1 and S2.

#### Leaching tests

Two representative samples (Htg1 and Htg6) were selected for metal(loid) fractionation studies to reflect the mineral composition of the material deposited at the waste pile. A sequential extraction procedure according to Tessier et al.^36^ was applied to partition trace metal(loid)s (Zn, Pb, Cd, Cu, As, Sb) into five fractions: exchangeable (F1), carbonate-bound (F2), Fe-Mn oxide-bound (F3), organic matter-bound (F4), and residual (F5). Additionally, leaching tests were conducted in accordance with EN 12457-2:2002 (European Committee for Standardization^37^) to evaluate the potential release of soluble constituents. This one-stage batch test was performed using material with a particle size below 4 mm, agitated for 24 h at a liquid-to-solid ratio (L/S) of 10 L/kg, employing deionized water as the leachant. ICP-ES determined the metal(loid) concentrations in the resulting extracts. A detailed summary of the operational parameters for each extraction step is provided in Table S3. The leaching test was conducted at the Laboratory of the Central Mining Institute – National Research Institute in Katowice, Poland.

## Results

### Bulk chemistry

The chemical composition of the flotation waste is dominated by Ca (mean 21.9 wt% CaO), Fe (mean 15.1 wt% Fe₂O₃), Mg (mean 9.73 wt% MgO), C (mean 8.43 wt% C), Si (mean 6.38 wt% SiO₂), S (mean 1.93 w% S), and Al (mean 1.47 wt% Al₂O₃) (Table 2). The samples also exhibited high Loss on Ignition values (mean 29.6 wt%) (Table 2). These results are consistent with the material’s characteristics and the gangue’s primarily dolomitic composition. Among the PTEs, the highest concentrations were observed for Zn (max. 11.2 wt%), Pb (max. 2.15 wt%), Cd (max. 1020 mg kg^− 1^), and As (max. 255 mg kg^− 1^) (Table 2). Noteworthy are also the elevated Ag concentrations, in some cases exceeding the method’s upper detection limit of 200 mg kg^− 1^ (Table 2). The samples exhibit very similar chemical compositions, both in terms of major and trace elements. Still, in the latter case, some samples show exceptionally high or low PTE values, which most likely result from local accumulations of minerals that serve as carriers of these elements (Table 2).

**Table 2:** Bulk chemical composition of ore-washing tailings from Tarnowskie Góry.

Sample no.		Htg1	Htg2	Htg3	Htg4	Htg5	Htg6	TG1	TG1.1	TG2	TG2.2	TG3	TG4	TG5	TG6	TG8	TG10
P_2_O_5_	wt%	0.05	0.05	0.03	0.07	0.06	0.04	na									
SiO_2_		4.57	6.78	3.68	8.35	9.31	5.58										
TiO_2_		0.03	0.06	0.02	0.07	0.08	0.03										
Al_2_O_3_		0.97	1.75	0.84	1.83	2.25	1.16										
Fe_2_O_3T_		12.80	15.40	13.40	18.30	15.70	15.00										
Cr_2_O_3_		< 0.01	< 0.01	< 0.01	< 0.01	< 0.01	< 0.01										
MnO		0.53	0.54	0.52	0.62	0.64	0.49										
CaO		26.40	23.80	22.40	21.70	18.80	18.30										
MgO		10.70	9.84	11.00	8.93	8.10	9.80										
Na_2_O		0.13	0.11	0.17	0.10	0.15	0.22										
K_2_O		0.07	0.16	0.08	0.15	0.20	0.11										
TOT/C		9.45	8.61	9.13	7.99	7.88	7.51										
TOT/S		0.13	1.42	2.20	0.16	2.08	5.60										
LOI		36.00	31.30	31.10	31.90	26.60	20.90										
Pb	mg kg^− 1^	7,880	8,540	7,170	11,500	21,500	10,300	8,140	3,146	> 10,000	9,460	> 10,000	> 10,000	12,106	> 10,000	7,600	> 10,000
Zn		52,500	44,400	82,500	39,600	84,700	112,000	> 10,000	> 10,000	> 10,000	> 10,000	> 10,000	> 10,000	24,413	> 10,000	> 10,000	> 10,000
Ag		61	63	83	55	122	119	26	2.8	151	3	86	76	18	> 200	> 200	61
As		93	141	155	119	148	255	3,870	2,453	405	3,330	154	181	460	920	114	231
Cd		433	431	617	572	951	1,020	538	132	1,014	137	500	416	31	105	19	342

na – not analyzed.

### Mineralogical composition and element speciation

**Fig. 2:**
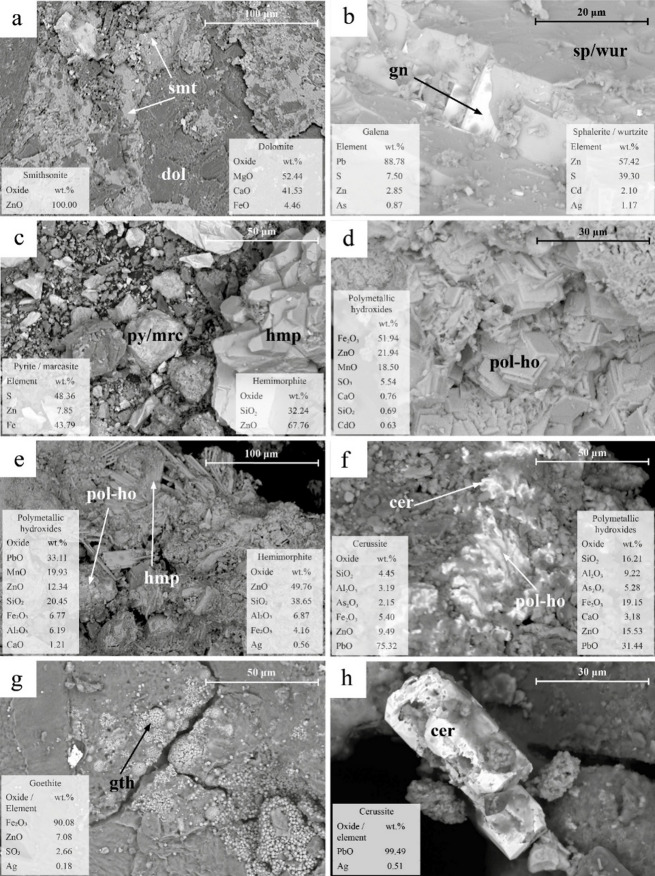
BSE images of ore-washing tailings. cer – cerussite; dol – dolomite; gn – galena; gth – goethite; hmp – hemimorphite; pol-ho – polymetallic hydroxides; py/mrc – pyrite/marcasite; smt – smithsonite; sp/wur – sphalerite/wurtzite.

Based on XRD (Table 3, Figure S1) and SEM–EDS analyses (Fig. 2), the tailings are largely composed of dolomite (average 65.5 wt%; CaMg(CO₃)₂), with subordinate amounts of goethite (average 10.4 wt%; α-Fe³⁺O(OH)), quartz (average 5.9 wt%; SiO₂), sphalerite/wurtzite (average 5.8 wt%; ZnS), and calcite (average 5.0 wt%; CaCO₃). Minor constituents comprise magnesite (MgCO₃), pyrite (FeS₂), hemimorphite (Zn₄Si₂O₇(OH)₂·H₂O), kaolinite (Al₂(Si₂O₅)(OH)₄), and cerussite (PbCO₃). Additionally, gypsum was detected in a single sample (Htg 6). The quantitative phase composition is broadly consistent across all ore-washing tailings, with variability occurring mainly at the qualitative level (Table 2).

**Table 3:** Phase composition of post-flotation tailings based on the Rietveld refinement of XRD data.

Sample no.		Htg1	Htg2	Htg3	Htg4	Htg5	Htg6
Dolomite - CaMg(CO_3_)_2_	wt%	79.4	69.8	46.1	63.6	78.7	55.1
Calcite - CaCO_3_		7.3	2.0	5.6	9.4	4.3	1.5
Quartz - SiO_2_		3.0	6.4	8.9	7.9	7.1	2.2
Goethite - α-Fe^3+^O(OH)		7.3	19.8	10.2	15.2	5.7	4.2
Hemimorphite - Zn_4_Si_2_O_7_(OH)_2_ · H_2_O		1.5	0.6	2.6	1.4	1.3	3.6
Kaolinite - Al_2_(Si_2_O_5_)(OH)_4_		0.2	0.3	2.5	1.0	0.1	-
Pyrite - FeS_2_		0.2	0.4	4.6	0.6	1.5	2.9
Sphalerite/wurtzite - ZnS		0.3	0.3	15.8	0.3	0.7	17.2
Cerussite - PbCO_3_		0.1	0.1	0.1	-	0.1	0.8
Magnesite- MgCO_3_		0.7	0.2	3.5	0.6	0.5	3.3
Gypsum - CaSO_4_ · 2H_2_O		-	-	-	-	-	9.0
		100.0	99.9	99.9	100.0	100.0	99.8

SEM–EDS analyses confirmed the phase assemblage identified by XRD. The ore-washing tailings are predominantly composed of dolomite aggregates (Fig. 2a), which reach several hundred micrometers in size and incorporate up to several percent FeO into their structure, approaching ankerite (Ca(Fe²⁺, Mg)(CO₃)₂). Among sulfides, sphalerite/wurtzite (Fig. 2b) is the dominant phase, occurring as anhedral to subhedral crystals up to several hundred micrometers in size. In contrast, subhedral galena and anhedral pyrite/marcasite (Fig. 2b, c), generally tens of micrometers across, are less abundant. These sulfides commonly contain Cd, As, and Ag in their structures, reaching concentrations of several wt%. (Fig. 2b, c).

Secondary mineralization is represented by irregular polymetallic (hydr)oxide aggregates of submicroscopic phases (Fig. 2d–f), which contain from several to several tens of percent of metal(loid)s such as Fe, Mn, Zn, Pb, As, and Cd. Goethite, typically enriched in a few wt% Zn was also identified in the tailings (Fig. 2g); however, its abundance in SEM observations does not account for the elevated concentrations indicated by XRD (Table 3). This suggests that the polymetallic (hydr)oxide aggregates likely contain significant amounts of goethite with additional metal(loid)s adsorbed onto its surfaces.

**Fig. 3:**
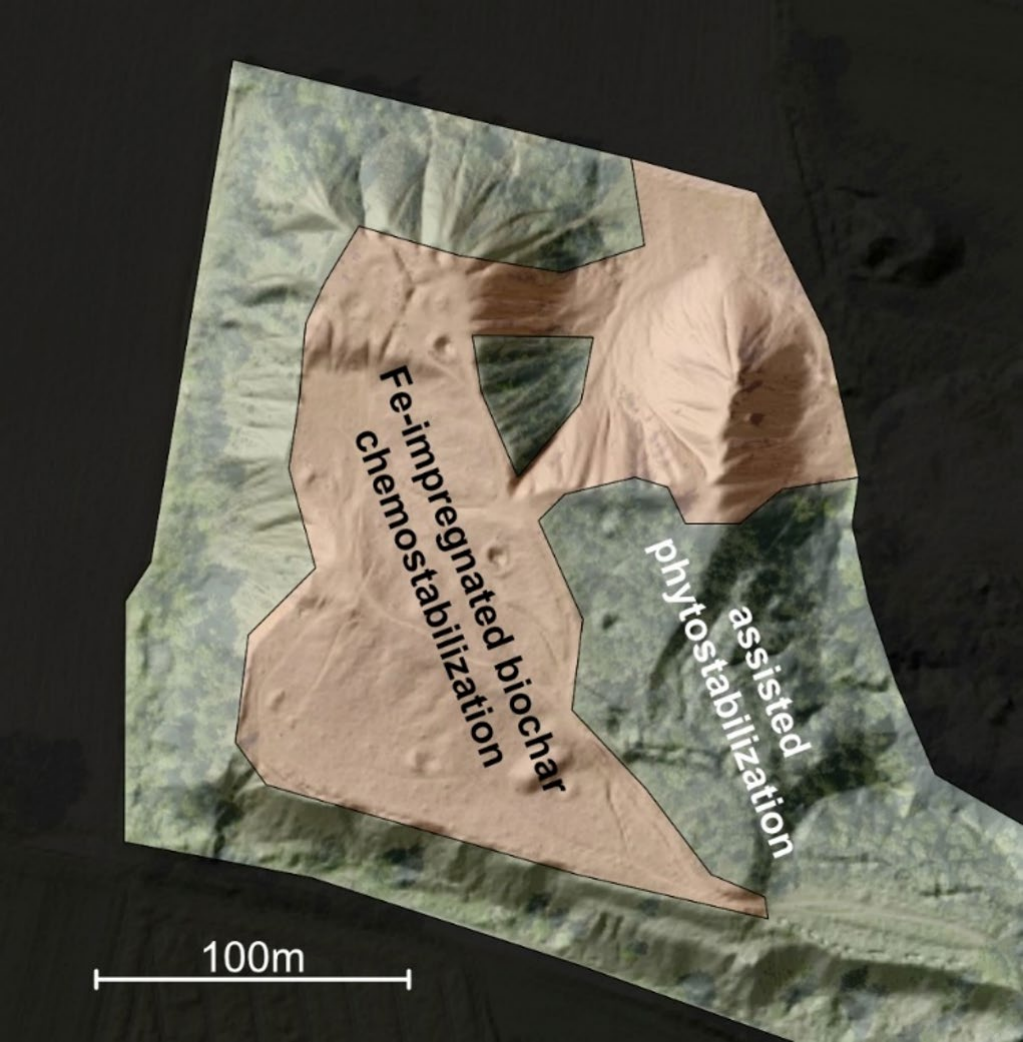
Satellite image with LIDAR data (geoportal.gov.pl) of Tarnowskie Góry heap with schematic illustration of dual-zone management concept.

**Table 4:** The results of leaching tests according to Tessier (1979) and EN 12457-2.

Element	Leaching stage according to Tessier (1979)					EN 12457-2
	mg kg^− 1^					
Sample Htg1						mg kg^− 1^
	F1	F2	F3	F4	F5	pH 7.8
As	< 1	< 1	< 1	< 1	90	0.05
Cd	27	127	52	3	< 1	0.05
Fe	10	6	2160	207	80,430	0.05
Pb	6	1970	2220	227	1140	0.05
Zn	83	10,090	4640	2510	7950	1.3
Sample Htg 6						pH 7.9
As	< 1	< 1	< 1	< 1	85	0.05
Cd	13	74	63	4	19	0.04
Fe	4	4	1430	164	95,400	0.74
Pb	12	2350	2050	208	1630	0.12
Zn	50	6750	4130	2330	8110	1.2

Based on the results, the primary zinc carrier in the ore-washing tailings is sphalerite/wurtzite, due to its high abundance in the material (Table 3). Hemimorphite is a secondary zinc host, followed by smithsonite to a lesser extent (Table 3; Fig. 2a, c, e). The amount of zinc in polymetallic (hydr)oxides is difficult to estimate, as it occurs at concentrations of several tens of wt%. (Fig. 2d, e, f). Within these aggregates, zinc may be present either as submicroscopic primary Zn minerals or adsorbed onto the structure of phases such as goethite.

Two primary lead minerals were identified in the tailings: galena, present in quantities too low to be detected by XRD, and cerussite (Table 3; Fig. 2f, h). Similar to zinc, lead is concentrated in polymetallic aggregates, with PbO content reaching several tens of wt% (Fig. 2).

Neither XRD nor SEM–EDS analyses detected As or Cd minerals in the tailings. However, EDS revealed that these elements are incorporated within the structures of other phases, primarily sulfides, polymetallic (hydr)oxides, and cerussite, at levels of up to a few wt% (Fig. 2).

Silver, in concentrations up to approximately 1 wt%, was detected in aggregates of galena and zinc sulfides (Fig. 2b), as well as within hemimorphite, goethite, and cerussite (Fig. 2e, g, h).

### Leaching tests

Leaching tests (sequential extraction and EN 12457-2) were performed on the tailings to evaluate the potential mobility of selected metal(loid)s under various conditions. The results from the leaching tests (Table 4) indicate that Fe and As are primarily associated with the residual fraction, suggesting a low potential for these elements to mobilize into the environment. For elements such as Cu, Tl, and Sb (Table S3), no increase in concentrations was recognized in any of the leaching steps. Their contents are often below the detection level of the method used.

Cadmium is the element of greatest concern regarding leachability from the tailings. Unlike most other metals, Cd was substantially partitioned into the readily releasable fractions (Table 4). Up to about 7–13% of the total Cd was extracted in the first, ion-exchangeable step (F1), and an additional ~ 40–60% was recovered in the weak-acid, carbonate-bound step (F2). These indicate that a significant amount of Cd is held in easily soluble forms. The absolute Cd concentration in the F1 leachate reached 13–27 mg kg^-1^, the highest relative fraction among the analyzed elements.

Zinc and lead demonstrate a more partitioned distribution across the extracted fractions (Table 4), indicating moderate mobility potential. A substantial portion of Zn in the tailings (up to ~ 40% of total Zn) was found in the carbonate-bound fraction (F2), with another sizable fraction (~ 30–40%) remaining in the residual phase (F5). A notable amount of Zn was also associated with iron/manganese oxide phases (F3), whereas only a minor amount (< 0.5%) occurred in the easily exchangeable form (F1) (Table 4). In absolute terms, the exchangeable Zn²⁺ released was on the order of 50–85 mg kg^-1^ of waste in the most extreme cases, given the high total Zn content (Table 4). Lead exhibits a broadly similar leaching profile, with the majority of Pb residing in relatively immobile fractions, primarily the carbonate (F2) and Fe/Mn oxide (F3) fractions. Only a minor amount of lead was bound to the exchangeable fraction (a few mg kg^-1^) (Table 4).

The tailings environment is highly buffered by carbonates, which directly affects leaching behavior. The waste material’s natural pH was determined to be alkaline (pH ~ 7.8–7.9) due to the abundance of dolomite, calcite, and ankerite in the tailings. Elevated pH favors the retention of metal cations by promoting their precipitation or sorption as insoluble carbonate and hydroxide phases. Consequently, despite Cd, Pb, and Zn at least partially occurring in easily leachable forms, the alkaline conditions significantly limit their actual mobilization, as confirmed by the low PTE concentrations in the leachate after testing with deionized water (Table 4).

## Discussion

### Environmental contamination risk and PTE mobility

The historical Zn–Pb–Ag waste heap at Tarnowskie Góry contains elevated concentrations of PTEs, notably Zn and Pb at percent levels and Cd at hundreds of mg kg^-1^, with As at lower concentrations (Table 2). Considering their average content in samples Htg1–6 (Table 2) and the estimated volume of the waste heap of 1.78 million tons, the dump in Tarnowskie Góry represents a reservoir of a substantial amount of PTEs: 123 000 t of Zn, 19 800 t of Pb, 1190 t of Cd, and 270 t of As. Interestingly, the heap also contains an estimated amount of 150 t of Ag.

Cadmium has a high leaching potential from the landfill, as indicated by the very high percentage of the ion-exchange fraction F1 (up to 13%) and the carbonate fraction F2 (up to 61%) (Table 4, Table S3). SEM/EDS studies allowed the identification of mineral phases enriched in Cd. Most commonly, these are polymetallic hydroxides and Zn-sulphides, with Cd concentrations up to 2.2 wt% (Fig. 2b, d). The importance of these findings lies in the fact that cadmium is a highly toxic metal for the environment, and its impact on biota is hazardous^25,17,38^. Cadmium concentrations in Zn-Pb ore washing waste are very high, often ranging from 10 to 1000 mg kg^-1^ (Table 2). High Cd contents were indicated by ores from the Silesian-Cracow region (Table 1), in soils contaminated by Zn-Pb smelting^39,40^ and slags^41,42^.The observed mobility of Cd, particularly when compared to Pb, results from distinct partitioning mechanisms within alkaline environments buffered by carbonates (pH ~ 7.9) ^43–45^. Mineralogical analyses revealed that Pb is predominantly partitioned into carbonates and polymetallic hydroxides, with lower amounts in sulfides (Fig. 2; Tables 3 and 4). Due to its very low aqueous solubility (Ksp = 10^-12.9^ to 10^-13.76^)^46,47^, cerussite acts as a stable, long-term sink for Pb. Cadmium precipitation, under similar conditions, could yield otavite (CdCO₃) ^45^, which has a solubility comparable to that of pure cerussite (Ksp of otavite is approx. 10^–12^); however, otavite rarely forms in natural environments ^43^. Instead, isomorphic substitution, facilitated by the similar ionic radii of Cd^2+^ and Ca^2+^, produces weatherable surface solid solutions with calcite that exhibit a relatively higher aqueous solubility (Ksp approx. 10^-8.48^) ^43,47^. The larger ionic radius of Pb^2+^ compared to Ca^2+^ in carbonate structures^48^ prevents its effective incorporation into the calcite lattice. This accounts for the high Cd concentrations found in the exchangeable and carbonate-bound sequential extraction fractions.

However, this high leachability in the F1 fraction contrasts sharply with the negligible Cd release observed in the standard EN 12457-2 water leaching test (Table 4). The extraction in F1 is driven by the high ionic strength of the 1 M MgCl₂ solution, where Mg^2+^ cations displace Cd^2+^ from mineral surfaces, while the abundant chloride anions form highly soluble complexes with the released cadmium (e.g., CdCl^+^). In contrast, the deionized water used in the EN 12457-2 test lacks both competitive ions and complexing ligands, failing to represent the complex chemistry of real-world infiltrates such as acid rain or organic-rich solutions. Furthermore, the EN 12457-2 test allows the dissolution of inherent carbonates and hydroxides in the tailings, which naturally buffer the leachate to a slightly alkaline pH (7.8–7.9).

Adsorption behaviors further differentiate the lability of Cd and Pb. Under the idealized conditions of the EN 12457-2 test, iron oxide surfaces (such as goethite) deprotonate at elevated pH and become negatively charged. This promotes the adsorption of Cd^2+^, effectively immobilizing the metal and rendering it highly insoluble in this aqueous environment. Pb^2+^, on the other hand, establishes stable inner-sphere covalent complexes with these iron oxyhydroxides. As a result, under identical conditions, Cd²⁺ demonstrates a lower specific affinity for these iron phases ^43^ and relies primarily on weaker outer-sphere electrostatic interactions, which are sufficient to limit mobility in pure water but are easily disrupted by extractants.

Zinc shows moderate mobility (Table 4) due to occurring within structure of stable minerals, i.e., sphalerite and wurcite (ZnS), smithsonite (ZnCO_3_), and hemimorphite (Zn_4_Si_2_O_7_(OH)_2_H_2_O) or adsorbed by iron hydroxodies (Table 4; Fig. 2). Hemimorphite and smithsonite are stable phases even in geological timescales^33^. Alkaline conditions (pH ~ 7.5–8.5, buffered by carbonate minerals) further modulate Zn mobility by promoting the precipitation of Zn-carbonates (smithsonite/hydrozincite) or adsorption onto iron hydroxides, thus self-limiting Zn concentrations in solution^44^. Limited mobility, together with the low toxicity of Zn to plants and biota ^10^, points to its low environmental impact within the investigated tailings.

Iron, though not a toxic element of concern itself, is present in very high amounts in the tailings (15.10 wt% of Fe_2_O_3_ on average; Table 2). It occurs predominantly as Fe^3+^ oxyhydroxides (primarily goethite; Table 3; Fig. 2) that were formed by oxidation of pyrite (FeS₂) and marcasite in the original ore and during waste weathering. Goethite imparts the red-orange coloration to the heap and is known for its stability under weathering conditions. Notably, Fe/Mn (hydr)oxides are known to act as scavengers for PTEs (e.g., Mamindy‑Pajany et al.^49^), and as mentioned earlier, they play a crucial role in the immobilization of Zn, Pb, and Cd in the Tarnowskie Góry heap (Fig. 2d, e, f; Table 4).

The Tarnowskie Góry heap has existed for over a century, allowing natural weathering processes to attenuate some environmental risks. The arid, carbonate-rich conditions have prevented the development of acid mine drainage (AMD), while in-situ secondary mineral formation has immobilized part of the PTEs. Thus, although the heap represents a considerable legacy source of contamination (Table 2), the current data suggest a relatively modest rate of PTE release into the environment (Table 4).

### Analogous sites and long-term environmental implications

The current state of limited contaminant release in Tarnowskie Góry is consistent with studies of historical mine wastes derived from other MVT deposits. For instance, the mine wastes from Plombières are characterized by a similar suite of PTEs (Pb, Zn, Cd, and As) and a comparable phase composition (mainly sulfides, carbonates, oxides, and hydroxides), which reflects the analogous nature of the exploited ore deposits (MVT type^50,51^). As in the studied material, the Plombières waste exhibits high concentrations of PTEs (e.g., Pb up to 51 800 mg kg^-1^, Zn up to 60 100 mg kg^-1^, Cd up to 429 mg kg^-1^, and As up to 3 170 mg kg^-1^), but moderate levels of PTE release during leaching tests under circumneutral pH conditions (6–8)^51^. Similarly, in another occurrence of MVT-type ores, the Raibl Zn–Pb district (NE Italy), researchers observed a comparable ease of element leaching as in the Tarnowskie Góry material. According to Barago et al.^52^, using 0.5 M HCl as the leaching agent, Cd was the most readily mobilized element, followed by Zn, Tl, and Pb, while As and Fe exhibited high stability. When comparing the concentrations of “extractable” elements between the Raibl district and Tarnowskie Góry, the former show markedly lower values (Table 4^52^), which, however, result from the use of a less selective leaching agent (1 M MgCl_2_ for F1 in Tarnowskie Góry vs. 0.5 M HCl for Raibl samples). When the values reported by Barago et al.^52^ are compared with the sum of fractions F1 and F2 (fractions that 0.5 M HCl should leach), the results are consistent in terms of both absolute emissions and the percentage of element mobilization. Importantly, concerning PTE mobility, the tailings from Tarnowskie Góry and Raibl exhibit similar natural pH values (~ 8). Although elevated concentrations of As, Cd, Pb, Tl, and Zn were detected in the Raibl district soils, two-thirds of them did not exceed the threshold limits for industrial and commercial sites specified in Italian law^52^.

The similarities between these sites and the studied material reinforce the conclusion that the Tarnowskie Góry waste heap, despite its currently limited contaminant release due to carbonate buffering, remains a long-term environmental liability. The PTEs sequestered within the pile can persist for centuries and may become mobile if site conditions change (e.g., soil acidification or mechanical disturbance). Contamination from ore mining can affect living organisms for millennia after mining ceases (e.g., Camizuli^19^.

### Site management challenges under UNESCO heritage constraints

The data provided reveal that the Tarnowskie Góry waste pile is a reservoir of PTEs. Although the abundance of carbonate gangue minerals provides a massive intrinsic neutralization potential^53^, which sequesters Pb and Zn mainly in stable secondary mineral phases, this stability is not uniform across all PTEs (Table 4). The critical management challenge identified in this study is the high lability of Cd, which, unlike Pb, does not form equally stable secondary phases and is prone to remobilization (Fig. 2, Table 4). This presents a specific vulnerability: natural pedogenic processes (e.g., acidification of the rhizosphere by plant root organic acids) could potentially strip Cd from the carbonate fraction, increasing its bioavailability to the vegetative cover. Therefore, management plans must focus on limiting Cd mobility while simultaneously maintaining the stability of Pb and Zn.

The inscription of the Tarnowskie Góry Lead-Silver-Zinc Mine and its Underground Water Management System on the UNESCO World Heritage List in 2017 fundamentally altered the trajectory of environmental management for the site. By recognizing the post-mining landscape not as a degraded wasteland but as a cultural asset of Outstanding Universal Value (OUV), the designation imposed a complex dual mandate: the preservation of the site’s physical integrity and authenticity must be reconciled with the imperative to mitigate latent environmental risks (Poland, UNESCO list, 2017). This creates a conservation-remediation paradox in which conventional engineering solutions employed to secure mine tailings, such as geometric reprofiling, capping with geosynthetic clay liners (GCL^54^), or ex-situ soil washing^55^, are rendered inapplicable. Such interventions would constitute a direct violation of UNESCO Criterion (iv), which cites the waste heap as a technological artifact illustrating the scale and efficiency of 19th-century ore processing (Poland, UNESCO list, 2017). Moreover, the legal framework governing the site, specifically the Local Protection Plan for the ‘Hałda Popłuczkowa’ Cultural Park^56^, establishes inflexible parameters for site interaction. As directly stated in the resolution: “The protection of the cultural park (…) is to be carried out by maintaining the heap complex in its unchanged present shape, along with the created soil layer and vegetation cover (…). In the area of the cultural park (…), it is prohibited to carry out earthworks distorting the terrain relief (…)”. This prohibition effectively rules out the use of thick isolation covers (cap-and-cover systems), which would obscure the historical “red earth” aesthetic derived from goethite-rich weathering products. Consequently, the management strategy must focus not on eliminating PTEs but on their in situ stabilization, which necessitates advanced, minimally invasive geochemical and ecological engineering approaches.

Given the dual constraints of Cd mobility together with high Pb, Zn concentrations, and strict landscape protection, assisted phytostabilization^57,58^ emerges as the most viable management technique by focusing on immobilizing metals in the root zone and reducing windborne dust dispersion, which is a primary exposure pathway for tourists. The colonization strategy should utilize native species of calamine grassland. Studies show that species such as Biscutella laevigata^59^ and Dianthus carthusianorum ^60^ are particularly suitable for land contaminated with Pb, Zn, and Cd. Additionally, Wierzbicka and Pielichowska^61^ have demonstrated that Biscutella laevigata possesses mechanisms to tolerate extreme Tl and Cd concentrations, stabilizing the upper soil horizon with a dense, shallow root system that effectively armors the surface against wind erosion. The use of deep-rooting trees (e.g., Betula pendula^62^) should be carefully managed or discouraged in specific zones, as their roots may penetrate the deeper, potentially sulphidic core of the heap, creating preferential flow paths for oxygen and water that could re-initiate oxidation processes and AMD. Areas subjected to assisted phytostabilization should be selected to meet criteria that preserve the visual character of the heap. The proposed conceptual zonation strategy (Fig. 3) directly addresses the conservation-remediation paradox: peripheral slopes, where visual impact is lower and which are already partially vegetated, can be managed with visible phytostabilization, while the historically sensitive core is treated with ‘invisible’ chemostabilization.

In locations where phytostabilization is unfeasible due to UNESCO regulations, it is essential to employ alternative methods for reducing Cd bioavailability, such as the application of stabilizing amendments. In this context, biochar appears to be the most promising solution; since it increases pH, it does not compromise the stability of secondary Pb and Zn phases, while being chemically stable and supplying organic functional groups for metal binding^63–65^. Research indicates that biochar alone can reduce extractable Cd in soils by approximately 32%, and by up to 52% when combined with zeolites^65^, owing to their high cation-exchange capacity^66^. Concurrently, studies clearly demonstrate that an amendment of merely a few percent (1–5%) of biochar is sufficient to achieve measurable effects^67,68^, confirming its economic viability and minimal impact on the visual character of the heap.

However, at the studied site, the application of standard biochar poses a significant risk of As remobilization^67,68^. The application of biochar may also introduce dissolved organic matter (DOM) into the environment, which actively displaces sequestered arsenic through competitive ligand exchange and can promote the reductive dissolution of Fe^3+^ to Fe^2+^. During this process, As bound to iron hydroxides may be released^69,70^. Moreover, DOM may also facilitate the reduction of As^5+^ to As^3+^, thereby increasing its toxicity and mobility^69^. Nevertheless, the increased mobility of As associated with biochar application can be mitigated by using engineered dual-function amendments, such as iron-impregnated biochar^71,72^. Produced by pyrolyzing biomass pre- or post-treated with iron phases (e.g., iron oxides or ferric salts), iron-impregnated biochar can effectively immobilize labile As while maintaining its capacity to retain Cd^69,71^. The incorporation of iron into the biochar matrix promotes the formation of highly reactive, amorphous iron phases, such as nanoscale goethite and ferrihydrite. These phases act as thermodynamic sinks, creating stable inner-sphere covalent complexes with arsenate, making them highly effective for arsenic adsorption^73^. However, given the site’s designation as a UNESCO World Heritage area, full-scale remediation requires prior long-term, site-specific testing.

Moreover, the strict prohibition on invasive drilling necessitates the deployment of non-destructive geophysical monitoring to assess the internal stability of the heap. The integration of Electrical Resistivity Tomography (ERT) and Induced Polarization (IP) provides a robust method for imaging the subsurface architecture. As evidenced by investigations at the analogous carbonate-hosted Raibl mine (Italy), ERT effectively delineates moisture content distribution and preferential flow paths, while IP chargeability contrasts can identify zones of disseminated sulfides and active oxidation^74,75^. Establishing a time-lapse ERT/IP monitoring program (e.g., on a 3–5-year cycle) would serve as an early warning system for internal geochemical instabilities, enabling precise, localized surface interventions that mitigate risk without compromising the authenticity of the post-mining landscape.

### Waste heap as metal(loid) inventory

Beyond its Outstanding Universal Value as a historical artifact, the Tarnowskie Góry waste heap constitutes a significant anthropogenic stock of metal(loid)s. Extrapolating bulk chemical analyses across the estimated 1.78 million tonnes of deposited material reveals substantial quantities of potentially useful metal(loid)s (Table 5). This is particularly significant given that tailings from Zn-Pb ore mining are often considered secondary sources for a broad spectrum of critical and base elements^76,77^. Currently, Zn and Ag represent the highest value among the identified metal(loid)s (Table 5). However, As deserves special attention, as it has been classified as a critical raw material under the EU Critical Raw Materials Act, underscoring its strategic importance and the need to secure and diversify its supply base^78^.

**Table 5:** Comparative summary of selected metal(loid)s from tailings in Tarnowskie Góry: estimated inventory, value, and key industrial applications.

Element	Est. Inventory (t)	2021–2026 Price (USD/t)	2021–2026 Est. value (million USD)	Key industrial applications
Zinc	123,000	~$2,250-~$4,500^1^	~ 277-~554	Zinc-based alloys, galvanization^79^
Lead	19,800	~$1,750-~$2,500^1^	~ 34.7-~49.5	Lead-acid battery industry^79^
Cadmium	1,190	~$2,560-~$4,100^79^	~ 3.05-~4.88	Ni-Cd batteries, alloys, coatings, and pigments^79^
Arsenic	270	~$2,450-~$4,500^79^	~ 0.66-~1.22	Chromated copper arsenate, GaAs semiconductors^79^
Silver	150	~$600,000-~2,950,000^2^	~ 90-~443	Electrical and electronics, photography, photovoltaics, coins and medals, jewelry^80^

^1^London Metal Exchange (https://www.lme.com/).

^2^London Bullion Market Association (https://www.lbma.org.uk/).

Although the exploitation potential must also be evaluated from the perspective of technological feasibility^76^, it should be noted that in the case of Tarnowskie Góry, UNESCO heritage preservation and the prohibition on landscape distortion effectively sterilize a mineral inventory estimated at $400 million to $1 billion (Table 5). Acknowledging this stockpile underscores the need for future policy frameworks capable of balancing intangible cultural preservation with the tangible recovery of critical raw materials demanded by the green transition.

### Limitations of the study and future perspectives

Several methodological and operational limitations of the presented study must be acknowledged.

The extraction fractions based on the Tessier et al.^36^ procedure function as operational proxies for element lability rather than direct measures of absolute field mobility. The high concentrations of Cd in the ion-exchangeable (F1) and carbonate-bound (F2) fractions reflect a susceptibility to mobilization under targeted dissolution constraints. As demonstrated by the negligible metal release in the EN 12457-2 leaching test using deionized water, the intrinsic buffering capacity of the prevailing dolomite and calcite matrix stabilizes the site’s macroscopic pH, inhibiting the bulk generation of AMD. At the same time, using deionized water as a leaching solution poorly reflects the complex chemistry of real-world environments. Consequently, the standard extraction protocols are limited in their ability to simulate realistic, localized environmental triggering mechanisms. The latent environmental risk at the Tarnowskie Góry site is highly conditional and resides explicitly at the microenvironmental scale. Future risk assessments and experimental work must address specific pathways capable of overcoming these localized buffering capacities, such as rhizosphere acidification driven by plant root exudates (e.g., low-molecular-weight organic acids) or episodic acidifying precipitation, which are inherently not captured by macroscopic bulk leaching assessments.

Estimating total metal stocks in the waste heap is hampered by sampling limitations, resulting in significant uncertainty. The volumetric totals (e.g., 123,000 t of Zn, 150 t of Ag) are based on extrapolating surface concentrations from waste samples to the entire estimated volume of 1.78 million tons. However, historical processing methodologies and subsequent supergene enrichment processes might have created spatial heterogeneity. Due to strict UNESCO heritage preservation mandates, invasive core drilling is legally prohibited. Therefore, the stated volumes represent indicative estimates rather than definitive resource classifications.

The proposed management strategy, particularly the application of iron-modified biochar and geophysical monitoring, is presented as a conceptual framework requiring empirical validation. Although theoretical models suggest that Fe-modified biochar can act as a dual-function sink to immobilize operationally labile Cd without triggering the secondary release of background As, a site-specific study is necessary.

Future work will focus on bench-scale validation, including column leaching and mesoscale experiments simulating seasonal wet-dry and redox cycles, to quantify potential side effects and long-term efficacy of the proposed strategy. While ERT and IP are proposed for non-destructive structural assessment, these methods yield indirect geophysical proxies. Their future deployment will require the calibration procedures to accurately correlate geophysical signatures with local biogeochemical variations.

## Conclusions

This study demonstrates that the management of UNESCO-listed mining sites requires a synergy between geochemical stabilization and heritage conservation. The mineralogical and leaching assessment of the Tarnowskie Góry tailings leads to the following management implications:

1. Geochemical constraints & cadmium risk: unlike acidic mine drainage scenarios, the carbonate-buffered environment (pH > 7.8) naturally immobilizes Pb and Zn. However, Cd represents a critical latent hazard due to its high exchangeability. Management plans must strictly prohibit acidifying vegetation (e.g., coniferous planting) that could trigger a pulse release of accumulated cadmium.
2. The engineered Fe-biochar solution for heritage integrity: conventional remediation (capping/thick vegetation) conflicts with the site’s Outstanding Universal Value by obscuring the industrial landscape. We propose that iron-modified biochar amendment is the optimal strategy for visually sensitive zones. While standard biochar immobilizes Cd, it risks mobilizing background arsenic. Theoretical models indicate that Fe-biochar prevents this by acting as a dual-function sink for both Cd and As, though formal bench-scale evaluation is required prior to field deployment. Moreover, it improves water retention to suppress dust while maintaining the bare-ground aesthetic required by heritage protection protocols, offering an invisible remediation alternative to phytostabilization.
3. Resource sterilization vs. preservation: current legal frameworks effectively sterilize significant metal resources (e.g., ~ 150 t Ag). Future heritage management policies should develop transparent frameworks to evaluate trade-offs between preserving intangible cultural values and the potential to recover critical raw materials.
4. Scalable management model: for carbonate-hosted mining legacies, we propose a two-zone management strategy: (1) peripheral slopes, where assisted phytostabilization is employed to control erosion, and (2) core historical features, where Fe-biochar-based chemostabilization is applied to preserve visual authenticity.

## Supplementary Information

Below is the link to the electronic supplementary material.

Supplementary Material 1

## Data Availability

Datasets generated are available in the Supplementary Materials file and are accessible in Zenodo data repository: https://doi.org/10.5281/zenodo.18350461.
